# ICOSLG-associated immunological landscape and diagnostic value in oral squamous cell carcinoma: a prospective cohort study

**DOI:** 10.3389/fcell.2023.1257314

**Published:** 2023-09-28

**Authors:** Yuexin Dong, Xinyang Hu, Shixin Xie, Yuxian Song, Yijia He, Wanyong Jin, Yanhong Ni, Zhiyong Wang, Liang Ding

**Affiliations:** ^1^ Central Laboratory of Stomatology, Affiliated Hospital of Medical School, Nanjing Stomatological Hospital, Nanjing University, Nanjing, China; ^2^ Department of Oral and Maxillofacial Surgery, Affiliated Hospital of Medical School, Nanjing Stomatological Hospital, Nanjing University, Nanjing, China

**Keywords:** ICOSLG, oral squamous cell carcinoma, prognosis, lymphocyte subsets, immune checkpoints

## Abstract

**Background:** We previously reported that stroma cells regulate constitutive and inductive PD-L1 (B7-H1) expression and immune escape of oral squamous cell carcinoma. ICOSLG (B7-H2), belongs to the B7 protein family, also participates in regulating T cells activation for tissue homeostasis via binding to ICOS and inducing ICOS^+^ T cell differentiation as well as stimulate B-cell activation, while it appears to be abnormally expressed during carcinogenesis. Clarifying its heterogeneous clinical expression pattern and its immune landscape is a prerequisite for the maximum response rate of ICOSLG-based immunotherapy in a specific population.

**Methods:** This retrospective study included OSCC tissue samples (n = 105) to analyze the spatial distribution of ICOSLG. Preoperative peripheral blood samples (n = 104) and independent tissue samples (n = 10) of OSCC were collected to analyze the changes of immunocytes (T cells, B cells, NK cells and macrophages) according to ICOSLG level in different cellular contents.

**Results:** ICOSLG is ubiquitous in tumor cells (TCs), cancer-associated fibroblasts (CAFs) and tumor infiltrating lymphocytes (TILs). Patients with high ICOSLGTCs or TILs showed high TNM stage and lymph node metastasis, which predicted a decreased overall or metastasis-free survival. This sub-cohort was featured with diminished CD4^+^ T cells and increased Foxp3+ cells in invasive Frontier *in situ*, and increased absolute numbers of CD3^+^CD4^+^ and CD8^+^ T cells in peripheral blood. ICOSLG also positively correlated with other immune checkpoint molecules (PD-L1, CSF1R, CTLA4, IDO1, IL10, PD1).

**Conclusion:** Tumor cell-derived ICOSLG could be an efficient marker of OSCC patient stratification for precision immunotherapy.

## 1 Introduction

Head and neck squamous cell carcinoma (HNSCC) is one of the most deadly cancers in the world. Oral squamous cell carcinoma (OSCC) is the most common cancer in HNSCC and has the characteristics of high metastasis rate and high recurrence rate (Yang et al., 2021).The treatment of OSCC is still mainly using traditional methods (surgery, chemotherapy, radiotherapy), unfortunately, this treatment mode is sticky to further improve the survival of OSCC patients (Chai et al., 2020).Tumor immunotherapy is a relatively novel treatment method, which has respectable prospects for controlling tumor recurrence and metastasis. Currently, the choice of immunotherapy for OSCC is extremely especially limited, so we urgently need more therapeutic targets to advance the survival and prognosis of OSCC patients (Almangush et al., 2021).

We previously found that stromal IL-33/ST2 signaling directly or indirectly enhanced PD-L1 (B7-H1)-mediated immune escape and OSCC progression (Ding et al., 2018; Zhao et al., 2023). ST2high/PD-L1high OSCC patients might be benefit more from anti-PD-1/L1 therapy. Notably, another immune checkpoint ICOSLG (ICOS ligand, B7-H2) is also a member of the B7 family, encoded by CD275, and is a ligand for the Inducible T cell co-stimulator (ICOS) (Greenwald et al., 2005; Henderson et al., 2011). In addition to the expression of professional antigen presenting cells (APCs, including B cells, macrophages, and dendritic cells), ICOSLG is also found in non-lymphocytes (including mesenchymal cells, vascular endothelial cells, Fibroblasts, tumor cells) in certain environments, such as tumor microenvironment. ICOSLG locates in the cell membrane and cytoplasm (Roussel and Vinh, 2021; Külp et al., 2022) and its interaction with ICOS can induce the production of various cytokines, thereby stimulating the differentiation and proliferation of T cells and promoting the activation of B cells (Zhang et al., 2023). Deletion or overexpression of ICOSLG may be associated with immune system diseases. Deletion may lead to T cell dysfunction-related diseases, such as Combined immunodeficiencies (CIDs) (Robertson et al., 2015; Roussel et al., 2018), and its overexpression may be associated with acute myeloid leukemia (AML) (Han et al., 2018).

As a co-stimulator of T cells, ICOSLG has also received much attention in the tumor microenvironment and its immune escape process (Zhang et al., 2022). It has been reported that high expression of ICOSLG promotes immunosuppression and tumor escape in gastric cancer by down-regulating function of Th1 type cells (Chen et al., 2003). In colorectal cancer, studies have found that increased expression of ICOSLG in CD8^+^ T cells leads to poor prognosis in patients (Cao et al., 2018). In patients with liver cancer, the high expression of ICOSLG is significantly associated with the recurrence and metastasis of patients, and the prognosis of positive patients is regularly poor (Zheng et al., 2019). In addition, in glioblastoma, high expression of ICOSLG promotes the proliferation of CD4^+^ T cells by stimulating the secretion of IL-10, but knocking out ICOSLG of tumor cells increases the number of CD8^+^ T cells (Iwata et al., 2020). However, the expression pattern and prognostic value of ICOSLG in OSCC remain unclear.

**FIGURE 1:**
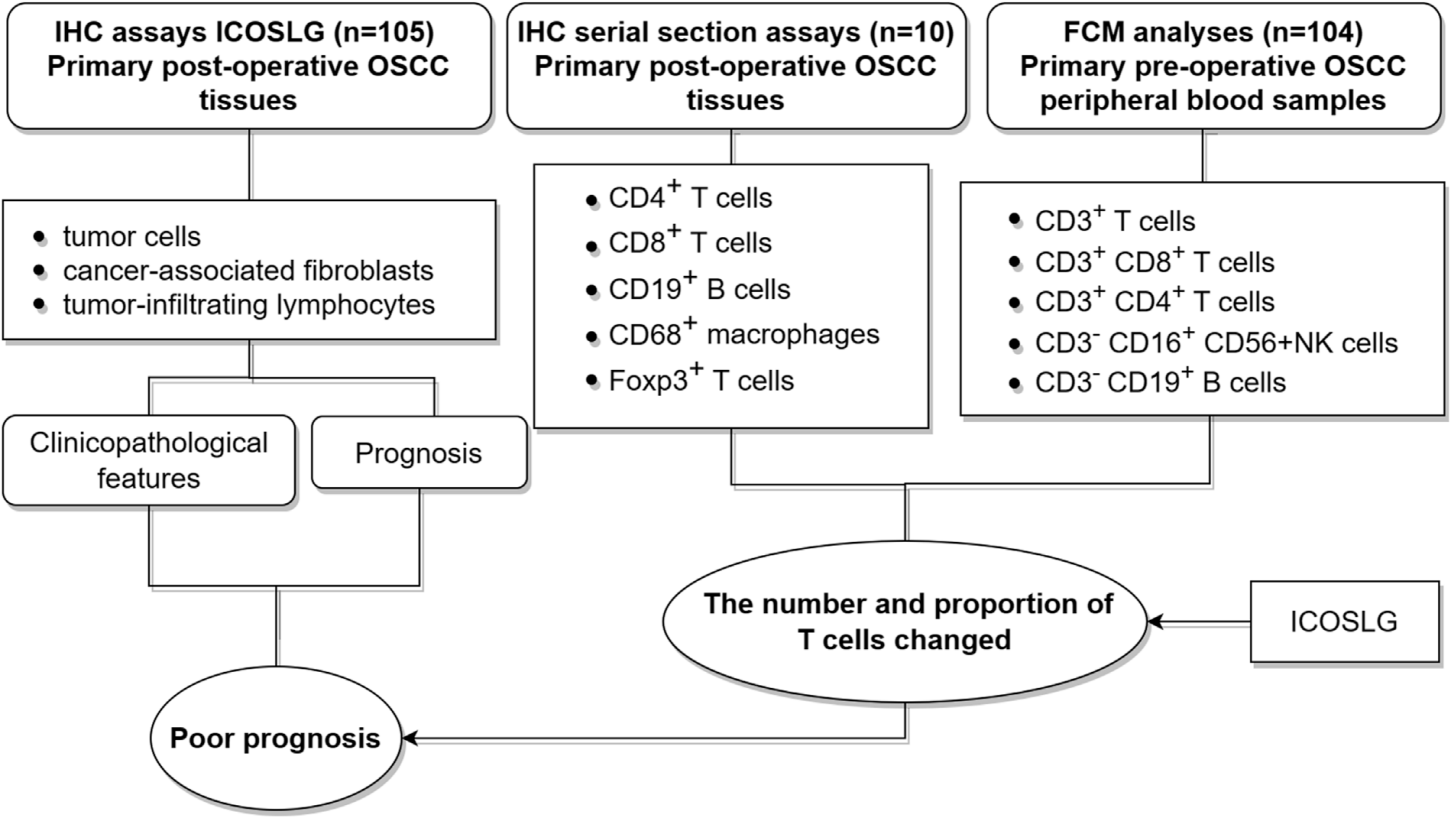
Experimental process design diagram.

In this study, we investigated the expression pattern of ICOSLG in oral squamous cell carcinoma, including tumor cells (TCs), cancer-associated fibroblasts (CAFs) and tumor infiltrating lymphocytes (TILs). Then the clinicopathological features of ICOSLG and its correlation with prognostic value were further analyzed. Considering the important role of ICOSLG in immune cells, we also analyzed the relationship between ICOSLG and lymphocyte subsets in peripheral blood and resident tissue misgovernment. In addition, considering that tumor cells can be used as the main mechanism of immune resistance through the immune checkpoint pathway, we further studied the association between ICOSLG and immune checkpoint molecules. The research concept of this paper is shown in Figure 1.

## 2 Materials and methods

### 2.1 Patients and samples

All the schemes of this study were examined and approved by the Ethics Committee of Nanjing Stomatology Hospital, Medical School of Nanjing University (No.2019NL-009(KS)). Informed consent was provided by the patients for the use of their tissues and data. The study was carried out in accordance with the Declaration of Helsinki. From 2014 to 2017, 105 primary OSCC patients were enrolled. The inclusion and exclusion criteria of patients were the same as those of our previous studies (Zhu et al., 2020). None of the patients received preoperative chemotherapy, radiotherapy, or other cancer-related treatments. Patients with history of systemic illness or missing survival data were excluded. These patients with primary tumor were diagnosed by hematoxylin and eosin staining by two experienced pathologists. These patients were followed up for 2–60 months, and the median was 38 months. Paraffin-embedded OSCC tissue slices were obtained from the pathology department and used for IHC study. 104 blood samples from OSCC patients were obtained for flow cytometry assay before any related treatments.

### 2.2 Immunohistochemistry and quantification

The protocol of IHC of formalin-fixed paraffin-embedded sections and scoring details of IHC was performed as previously described (Zhao et al., 2019). Anti-ICOSLG (ab257321, Abcam, Waltham, MA, USA) were used at a dilution ratio of 1:400, and the serial sections were incubated with primary antibodies such as anti-CD4 (ZSGB-BIO, ZM-0418), anti-CD8 (ZSGB-BIO, ZA-0508), anti-CD19 (ZSGB-BIO, ZM-0038), anti-CD56 (ZSGB-BIO, ZM-0057), anti-CD68 (ZSGB-BIO, ZM-0464), and anti-Foxp3 (ab253297, Abcam). We used PBS to replace the primary antibody as negative control. Due to insufficient tissues and individual differences in OSCC samples, certain regions or cell types, such as CAFs and TILs, could not be detected in the IHC staining.

Protein expression was evaluated according to stain intensity and the percentage of positive cells. The intensity of staining was graded as 1 = weak staining, 2 = moderate staining and 3 = strong staining. The percentage of stained cells was graded as 0 = 0–5%, 1 = 6–25%, 2 = 26–50%, 3 = 51–75% and 4 = 75–100%. The final score was obtained by multiplying the two scores. The expression levels of ICOSLG in TCs and CAFs, TILs were defined as “low” when it is lower than the median value and as “high” when it is equal to or greater than the median. The IHC staining results of ICOSLG were evaluated by two senior pathologists who did not know the patient’s data, and the median values were calculated for further analysis.

### 2.3 Preparation of PBMC

Fresh whole blood from patients was collected with EDTA tube (ethylenediamine tetraacetic acid tube, BD Vacutainer). On average, 1.0 × 107 cells were isolated from 5 mL of whole blood. The collected whole blood was 2× diluted with Hanks’ Balanced Salt solution (HBSS, Gibco, Rockville, MD, USA) for loading on Ficoll-paque (Pharmacia, Uppsala, Sweden). The blood-loaded sample on Ficoll solution is centrifuged at 2000 rpm for 20 min (Acceleration/Break = lowest/zero), and middle layer was collected as PBMC. The collected cells were enumerated and stored in liquid nitrogen tank until us. All study participants provided informed consent, and was approved by the ethical committee of Nanjing Stomatology Hospital, Medical School of Nanjing University.

### 2.4 Flow cytometry assay

For the cell subtypes of PBMC analysis, cells were collected and washed with PBS twice and then suspended in 200 μL PBS. For enumeration of mature human T (CD3^+^) cells, helper/inducer T (CD3^+^ CD4^+^) cells, cytotoxic T (CD3^+^ CD8^+^) cells, B (CD19^+^) cells, and NK (CD3^−^CD16^+^and/orCD56^+^) lymphocytes, CD3^−^FITC/CD8^−^PE/CD45^−^PerCP/CD4^−^APC reagent, BD Multitest CD3^−^FITC/CD16^−^PE^+^ CD56^−^PE/CD45^−^PerCP/CD19^−^APC reagent were used according to the manufacturer’s instructions, respectively (Cat No.340503, BD Multitest™), then quantified by flow cytometry on a FACS Calibur instrument.

### 2.5 Gene correlation analysis in cBioPortal

The cBioPortal for Cancer Genomics (http://cBioPortal.org) is a website for exploration of multi-dimensional cancer genomics data, providing readily understandable gene expression event (Gao et al., 2013). We used cBioPortal to analyze the correlation between ICOSLG and specific lymphocyte subset markers as well as specific immune checkpoint molecules in HNSCC. Co-expression was calculated based on the cBioPortal’s online instructions.

### 2.6 Tisch2 analysis

Tumor Immune Single-cell Hub 2 (Tisch2, http://tisch.comp-genomics.org) is a scRNA-seq database focusing on tumor microenvironment (TME). TISCH2 provides detailed cell-type annotation at the single-cell level, enabling the exploration of TME across different cancer types. We used TISCH2 to evaluate the difference in ICOSLG between tumor cells and normal cells in different tumors. In addition, according to the online description of Tisch2, we also evaluated the correlation between ICOSLG and specific immune infiltrating cell subsets at the transcriptional level.

### 2.7 Statistical analysis

SPSS 18.0 and GraphPad Prism 8.0 software packages were used for data analysis and graphic processing. Pearson’s chi-square test, Fisher’s exact test and Chi-square test were used to compare clinicopathological features. The Mann–Whitney U test was used to compare the two groups. Survival analysis includes overall survival (OS), metastasis-free survival (MFS) and disease-free survival (DFS), which were evaluated by Kaplan–Meier and log-rank test. Further multivariate analysis was carried out by Cox proportional hazards regression model to determine the independent risk factors, adjusted hazard ratio (HR) and 95% confidence interval (CI) of OSCC. Co-expression between ICOSLG and immune cell markers and immune checkpoint molecules was investigated by Pearson correlation analysis. The partial Spearman’s correlation analysis was used to analyze the association between ICOSLG and markers of specific immune infiltrating cell subset at transcription level. All statistical tests were two-sided, and *p* < 0.05 was considered to be significant.

## 3 Results

### 3.1 ICOSLG is widely expressed in TC, CAF and TIL in OSCC

**FIGURE 2:**
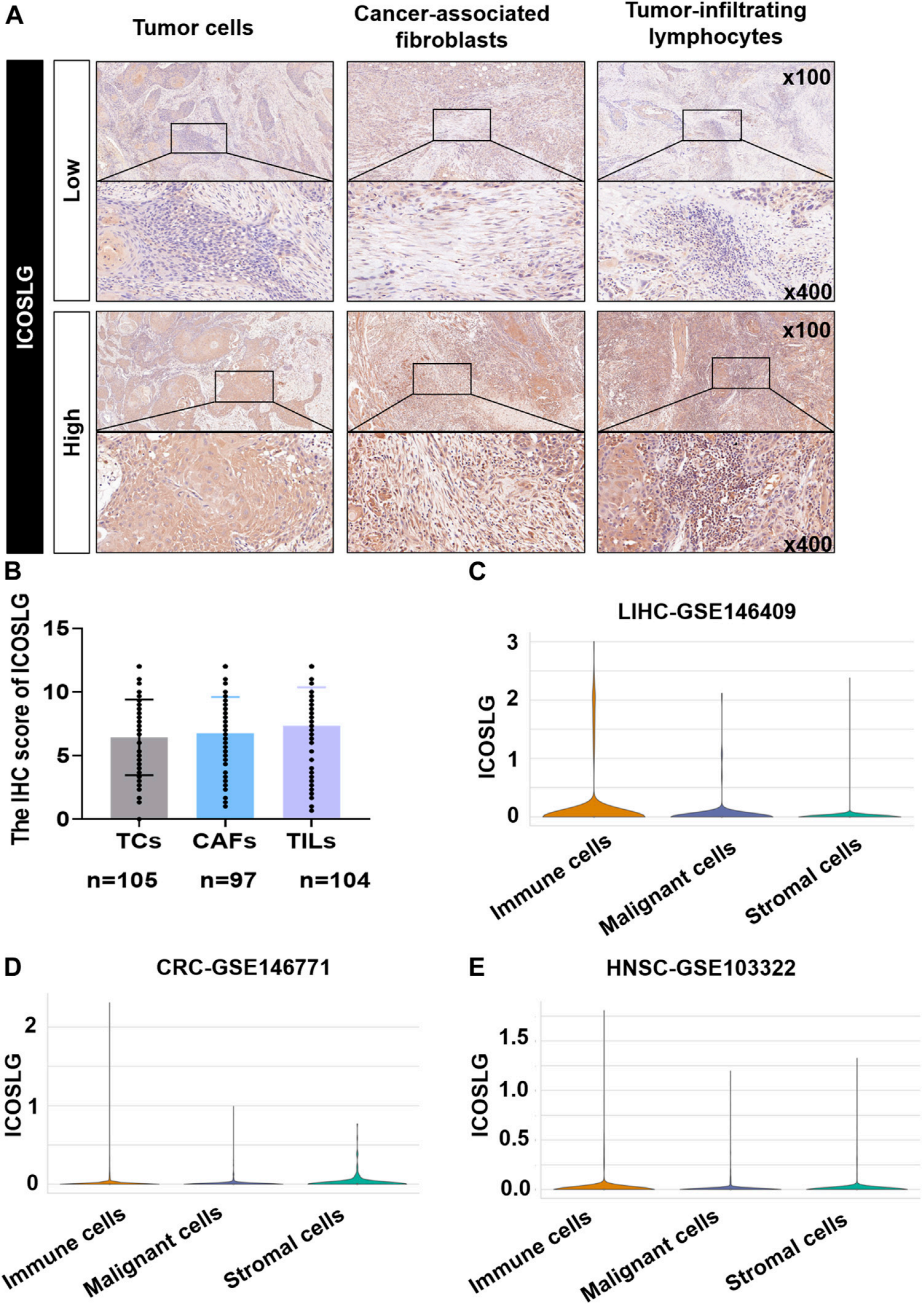
The expression of ICOSLG in OSCC and other tumors. **(A)**. Typical IHC staining of ICOSLG on TCs, CAFs and TILs. **(B)**. The IHC score of ICOSLG in TCs, FLCs, and TILs from OSCC patients. The ICOSLG expression in liver hepatocellular carcinoma **(C)**, colorectal cancer **(D)**, and head and neck squamous cell carcinoma **(E)** with Tisch2.

ICOSLG was expressed in the cell membrane and cytoplasm of tumor cells (TCs), cancer-associated fibroblasts (CAFs) and tumor infiltrating lymphocytes (TILs) in 105 patients with OSCC. The typical low expression and high expression of ICOSLG IHC staining were shown in Figure 2A. In OSCC, the IHC score of TILs was generally higher than that of TCs. (Figure 2B). In the TISCH2 database, single-cell sequencing analysis showed that the mRNA expression of ICOSLG was significantly higher in TILs in liver hepatocellular carcinoma (Figure 2C), colorectal cancer (Figure 2D), and head and neck squamous cell carcinoma (Figure 2E).

### 3.2 Patients with high ICOSLG have a higher risk of TNM stage and lymph node metastasis

**FIGURE 3:**
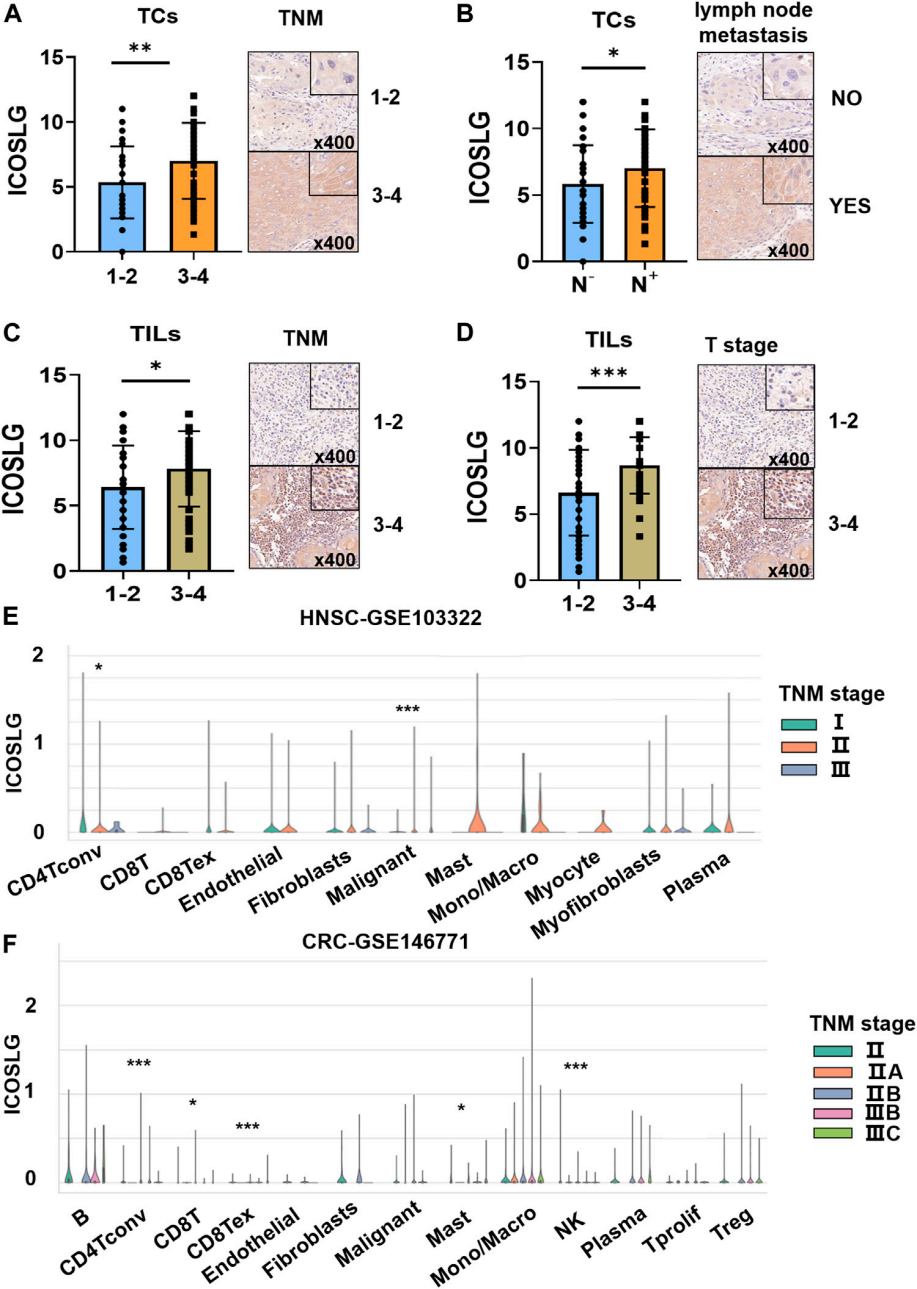
The relationship between ICOSLG and clinicopathological parameters. ICOSLG expression with different TNM stages **(A)** and lymph node metastasis **(B)** in TCs, different TNM stages **(C)** and T stages **(D)** in TILs. Tisch2 database was used to detect the relationship between the expression of ICOSLG in different cells and TNM stage in colorectal cancer **(E)** and head and neck squamous cell carcinoma **(F)**. *, **, *** represented that differences were considered statistically significant with *p* < 0.05, *p* < 0.01 and *p* < 0.001 respectively, and ns represented no statistical differences.

We then analyzed the relationship between the expression of ICOSLG and clinicopathological features of OSCC patients (Table 1). The results showed that in OSCC patients, the expression of ICOSLG was not significantly correlated with gender, age, and differentiation, but the high expression of ICOSLG in TCs (ICOSLGTCs) and TILs (ICOSLGTILs) was associated with higher risk of lymph node metastasis and advanced TNM stage. IHC results showed that high expression of ICOSLG in TCs (ICOSLGTCs) was associated with higher risk of lymph node metastasis (Figure 3A) and advanced TNM stage (Figure 3B), while ICOSLG in TILs (ICOSLGTILs) was significantly associated with advanced TNM stage (Figure 3C) and T stage (Figure 3D).We analyzed the Tisch2 database and found that in patients with head and neck squamous cell carcinoma, the high expression of ICOSLGTCs was significantly positively correlated with advanced TNM stage, while the high expression of ICOSLGTILs, especially CD4^+^ T cells, was negatively correlated with advanced TNM stage (Figure 3E). The high expression of ICOSLG on CD4^+^ T cells is positively correlated with advanced TNM stage in colorectal cancer (CRC) (Figure 3F).

**TABLE 1:** Association between ICOSLG expression and clinicopathological characteristics in OSCC patients.

**Characteristics**	**TCs**					**CAFs**					**TILs**				
	**Total**	**Low**	**High**	**χ2**	***P***	**Total**	**Low**	**High**	**χ2**	***P***	**Total**	**Low**	**High**	**χ2**	***P***
**Gender**															
Female	43	21 (48.8%)	22 (51.2%)	0.138	0.71	38	19 (50%)	19 (50%)	0.06	0.807	43	22 (51.2%)	21 (48.8%)	0.28	0.597
Male	62	28 (45.2%)	34 (54.8%)			59	28 (47.5%)	31 (52.5%)			61	28 (45.9%)	33 (54.1%)		
**Age**															
<60	32	17 (53.1%)	15 (46.9%)	0.771	0.38	31	16 (51.6%)	15 (48.4%)	0.182	0.67	32	17 (53.1%)	15 (46.9%)	0.472	0.492
≥60	73	32 (43.8%)	41 (56.2%)			66	31 (47%)	35 (53%)			72	33 (45.8%)	39 (54.2%)		
**TNM**															
Ⅰ-Ⅱ	37	23 (62.2%)	14 (37.8%)	5.512	0.019*	32	20 (62.5%)	12 (37.5%)	3.772	0.052	37	24 (64.9%)	13 (35.1%)	6.484	0.011*
Ⅲ-Ⅳ	68	26 (38.2%)	42 (61.8%)			65	27 (41.5%)	38 (58.5%)			67	26 (38.8%)	41 (61.2%)		
**T stage**															
1–2	69	36 (52.2%)	33 (47.8%)	2.452	0.117	62	34 (54.8%)	28 (45.2%)	2.805	0.094	69	38 (55.1%)	31 (44.9%)	4.019	0.045*
3–4	36	13 (36.1%)	23 (63.9%)			35	13 (37.1%)	22 (62.9%)			35	12 (34.3%)	23 (65.7%)		
**Lymph node metastasis**															
No	53	32 (60.4%)	21 (39.6%)	8.083	0.004*	48	29 (60.4%)	19 (39.6%)	5.445	0.020*	52	31 (59.6%)	21 (40.4%)	5.547	0.019*
Yes	52	17 (32.7%)	35 (67.3%)			49	19 (38.8%)	31 (61.2%)			52	19 (36.5%)	33 (63.5%)		
**Differentiation**															
Well	21	6 (28.6%)	15 (71.4%)	3.453	0.063	20	9 (45%)	11 (55%)	0.12	0.729	21	7 (33.3%)	14 (66.7%)	2.291	0.13
Moderate to poor	84	43 (51.2%)	41 (48.8%)			77	38 (49.4%)	39 (50.6%)			83	43 (51.8%)	40 (48.2%)		

TCs, tumor cells; CAFs, cancer‐associated fibroblasts; TILs, tumor‐infiltrating lymphocytes; χ2, Pearson’s chi‐squared test.* represented that differences were considered statistically significant with P < 0.05.

### 3.3 High ICOSLG level in tumor cell and TILs predicts low survival rate of OSCC patients

**FIGURE 4:**
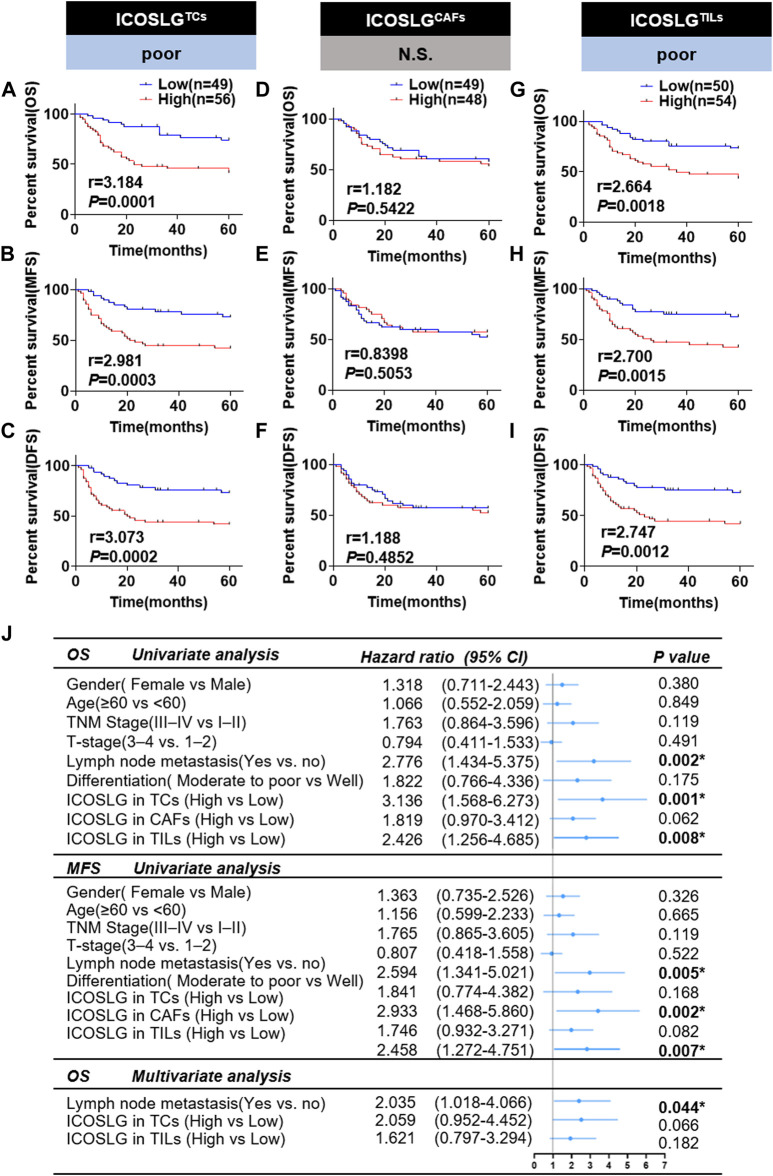
The relationship between ICOSLG and the prognosis of OSCC patients. Kaplan-Meier survival curve of overall survival time (OS), metastasis-free survival time (MFS) and disease-free survival time (DFS) of OSCC patients, according to the expression of ICOSLG in TCs **(A–C)**, CAFs **(D–F)** and TILs **(G–I)**. Cox-regression analysis and forest plot of OS and MFS in OSCC patients **(J)**. CI, confidence interval; ICOSLGTCs, ICOSLG in TCs; ICOSLGCAFs, ICOSLG in CAFs; ICOSLGTILs, ICOSLG in TILs. * represented that differences were considered statistically significant with *p* < 0.05, N.S. represented no significance.

In order to confirm the prognostic value of ICOSLG for OSCC, we used Kaplan-Meier survival rate to analyze the survival rate of patients with oral squamous cell carcinoma included in this study. The results showed that patients with increased ICOSLG expression in TCs had shorter overall survival (OS r = 3.184), metastasis-free survival (MFS r = 2.981) and disease-free survival (DFS r = 3.073) (Figures 4A–C). In addition, OSCC patients with more ICOSLG in TILs had shorter OS (r = 2.664), MFS (r = 2.700) and DFS (r = 2.747) (Figures 4G–I), which was not observed in ICOSLG in CAFs (ICOSLGCAFs) (Figures 4D–F).

We used univariate and multivariate Cox regression to analyze the prognostic value of clinicopathological features. The results showed that gender, age, TNM stage, T stage, differentiation and ICOSLG in CAFs (ICOSLGCAFs) had no significant predictive value for OS and MFS (all *p* > 0.05). Lymph node metastasis and high expression of ICOSLG in TCs and TILs were significantly different in OS and MFS, but not independent prognostic indicators of oral squamous cell carcinoma (Figure 4J).

### 3.4 Resident tissue CD4^+^ T cells show an exhausted trend in patients with high ICOSLG^TCs or TILs^

**FIGURE 5:**
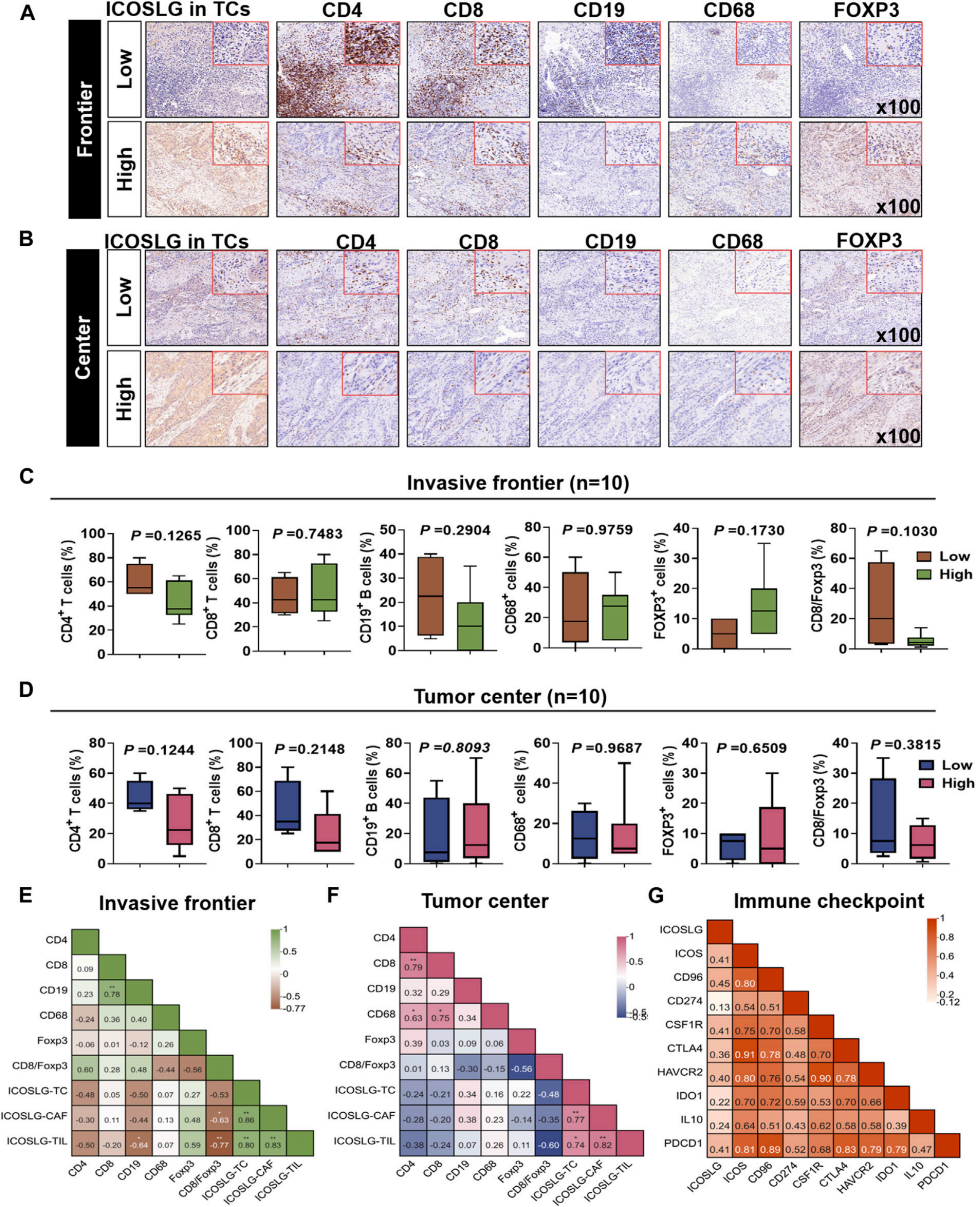
The proportion of different immune cells was affected by ICOSLG. The correlation between ICOSLG expression and CD4^+^ T cells, CD8^+^ T cells, CD19^+^ B cells, CD68^+^ TAMs and FOXP3+ Tregs in OSCC tumor invasive Frontier **(A)** and tumor center **(B)** serial sections with immunohistochemistry. The proportion of the above cells in the tumor invasive Frontier **(C)** and tumor center **(D)** was compared between the ICOSLG high expression group and the low expression group, as well as the intra-group correlation analysis with ICOSLGTC, ICOSLGCAF, ICOSLGTILs **(E,F)**. Intra-group correlation analysis between ICOSLG expression and immune checkpoints in HNSCC with cBioPortal database **(G)**.

To explore whether the immune cell subsets of tumor tissues with high expression of ICOSLGTCs in OSCC patients changed, we determined the ICOSLG, CD4, CD8, CD19, CD68 and Foxp3 level of tumor centers and infiltration fronts in serial sections (Figures 5A,B). Next, we compared the proportion of CD4, CD8, CD19, CD68 and Foxp3 positive cells in lymphocytes of patients with high and low expression of ICOSLGTCs, as well as the proportion of CD4, CD8 positive cells and Fopx3+ cells, and divided them into invasive Frontier (Figure 5C) and tumor center (Figure 5D) for analysis. CD4^+^ cells and CD19^+^ cells in patients with high expression of ICOSLGTCs at the invasive Frontier showed a decreasing trend, while Foxp3+ cells showed an increasing trend. However, CD4^+^ cells and CD8^+^ cells in patients with high expression of ICOSLGTCs at the tumor center showed a decreasing trend.

We also analyzed the ICOSLGTILs-regulated in the intra-group correlation comparison of CD4, CD8, CD19, CD68, Foxp3, CD8/Foxp3, ICOSLGTCs, ICOSLGCAFs and ICOSLGTILs, we found that ICOSLGTILs were negatively correlated with CD4 and CD19 in the invasive Frontier (Figure 5E), while ICOSLGTILs were positively correlated with Foxp3 and ICOSLGTCs. In the tumor center (Figure 5F), ICOSLGTCs and ICOSLGTILs were negatively correlated with CD4 and CD8. Immune checkpoint proteins play a crucial role in the negative regulation of cellular immunity. Therefore, we used cBioPortal to further analyze the correlation between ICOSLG and immune checkpoint molecules, and analyzed the correlation between immune checkpoints (Figure 5G). We found that ICOSLG was positively correlated with inducible T cell co-stimulator (ICOS r = 0.403), TACTILE (CD96 r = 0.444), programmed death-ligand 1 (CD274 r = 0.124), colony-stimulating factor 1 receptor (CSF1R r = 0.409), cytotoxic T lymphocyte antigen 4 (CTLA4 r = 0.356), hepatitis A virus cellular receptor 2 (HAVCR2 r = 0.404), indoleamine 2,3-dioxygenase 1(IDO1 r = 0.224), interleukin 10 (IL10 r = 0.247), programmed cell death 1 (PDCD1 r = 0.400), and these immune checkpoints were also positively correlated.

### 3.5 CD4^+^ and CD8^+^ T cells in peripheral blood of patients with high expression of ICOSLGTCs are also significantly reduced

**FIGURE 6:**
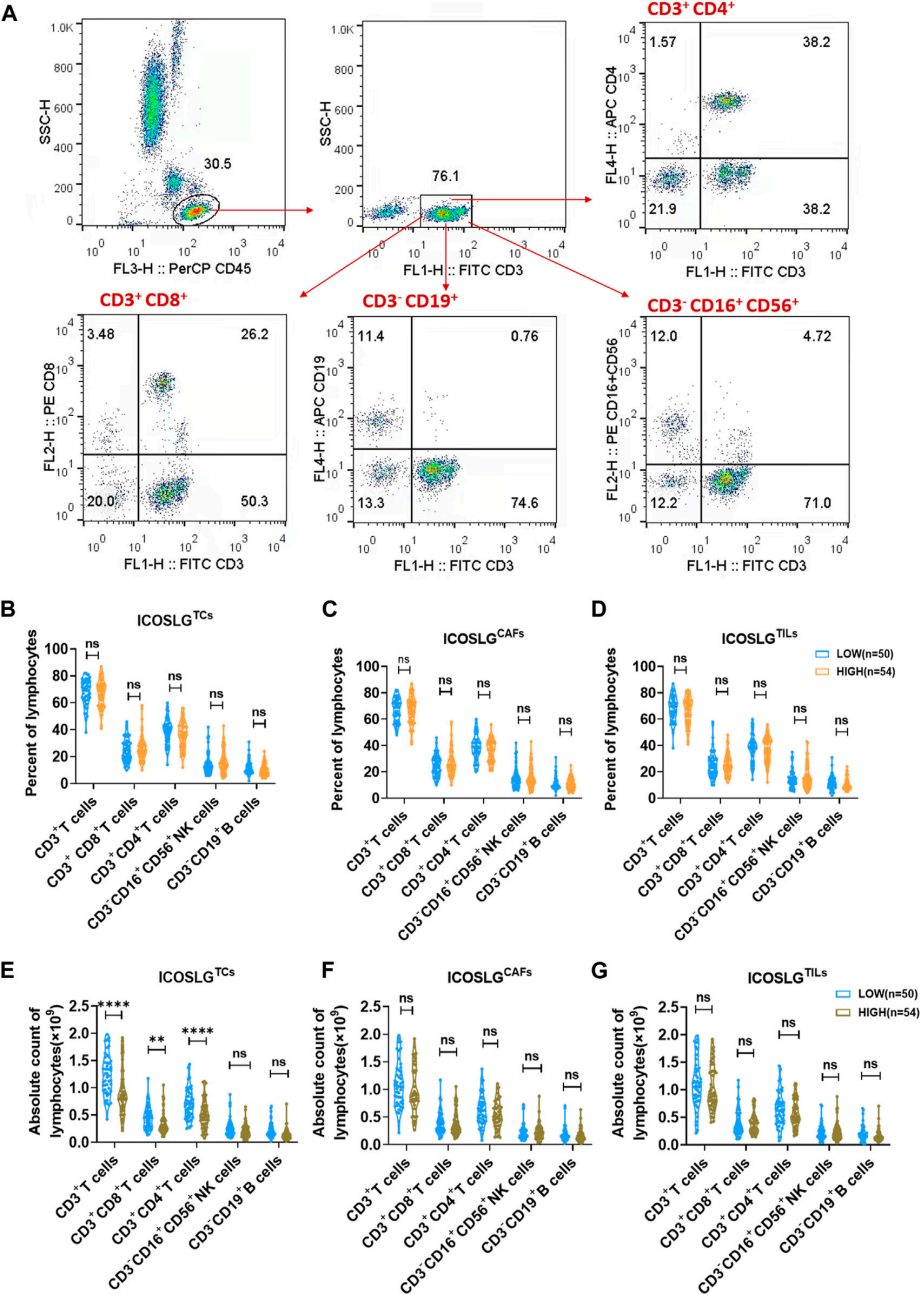
The change of lymphocytes subset in PBMC and tissue of OSCC patients according to ICOSLG level. **(A)**: Flow-cytometry dot plots show the strategy for gating lymphocytes, CD3^+^ T cells, CD3^+^CD4^+^ T cells and CD3^−^CD16^+^CD56^+^ NK cells with distinct expression of ICOSLG TILs. The radio of lymphocytes subset of PBMCs in patients with distinct expression of ICOSLGTCs **(B)**, ICOSLGCAFs **(C)**, ICOSLGTILs **(D)**. The absolute count of lymphocytes subset of PBMCs in patients with distinct expression of ICOSLGTCs **(E)**, ICOSLGCAFs **(F)**, ICOSLGTILs **(G)**. **, **** represented that differences were considered statistically significant with *p* < 0.01, *p* < 0.0001 respectively, and ns represented no statistical differences.

IHC serial sections of OSCC patients showed a correlation between ICOSLG and the number of CD4^+^, CD8^+^, CD19^+^, Foxp3+ cells *in situ*. Therefore, we used flow cytometry to analyze the proportion of peripheral blood T, B and NK cells between the low ICOSLG group and the high ICOSLG group, and the strategy of gating lymphocytes was shown in Figure 6A. We found that the absolute counts of CD3^+^ T cells, CD3^+^ CD4^+^ T cells and CD3^+^ CD8^+^ T cells in ICOSLGTCs high expression samples were significantly lower (Figure 6E). However, there was no difference in the percentage of lymphocyte subsets in ICOSLGTCs (Figure 6B) and the frequency and number of lymphocyte subsets in ICOSLGCAFs (Figures 6C, F), ICOSLGTILs (Figures 6D, G) between the low and high subgroups of ICOSLG.

## 4 Discussion

ICOSLG was found in monocyte-derived dendritic cells, initial studies of ICOSLG focused on immune cells, its co-expression with ICOS can regulate the activation of CD4^+^ T cells (Wang et al., 2000; Wallin et al., 2001; Witsch et al., 2002).At present, with the expansion of research, ICOSLG has been confirmed to be associated with a variety of diseases, including immunodeficiency diseases (Dalakas, 2005), hematological diseases (Tamura et al., 2005). In addition, ICOSLG is expressed in a variety of tumors, such as glioblastoma (Schreiner et al., 2003), gastric cancer (Chen et al., 2003), colorectal cancer (Xiao et al., 2005), etc. The results of this study showed that ICOSLG was widely expressed in OSCC samples and was significantly expressed. Therefore, we conclude that ICOSLG is actually involved in the occurrence and development of tumors, and can regulate the behavior of tumor cells and affect the changes of tumor microenvironment.

Current studies have shown that the expression of ICOSLG in a variety of tumors is related to the biological behavior of tumors and the poor prognosis of patients. In melanoma, the high expression of ICOSLG on tumor cells is closely related to the decrease of patient survival. In addition, in gastric cancer, co-stimulation of ICOS and ICOSLG can lead to the activation of Tregs cells and may be associated with poor prognosis of patients (Nagase et al., 2017). According to our analysis, in OSCC, the high expression of ICOSLG in TCs and TILs was related to TNM stage and lymph node metastasis, and the OS, MFS and DFS of patients with high expression of ICOSLG in TCs and TILs were significantly shorter, indicating that the high expression of ICOSLG in OSCC may be a poor prognostic indicator. However, it should be noted that the high expression of ICOSLG in TCs and TILs is not an independent prognostic factor for OSCC.

ICOSLG is closely related to the functional activation of T cells. In the tumor microenvironment, it plays an important role in the development of tumors by participating in the regulation of immune cell function. In breast cancer, the activation of ICOS and ICOSLG is closely related to the accumulation of Tregs cells and DCs, and is also an indispensable key factor in the activation of Tregs cells (Faget et al., 2012). In esophageal squamous cell carcinoma, inhibition of ICOSLG can reduce the number of Tregs cells, thereby improving the effect of tumor immunotherapy (Zhang et al., 2022). In myeloma, tumor cells with high expression of ICOSLG have stronger proliferation ability and can activate ICOS-ICOSLG pathway to inhibit tumor immune response (Yamashita et al., 2009). Our results also found that in OSCC, the high expression of ICOSLG in TCs led to a decrease in CD4^+^ T cells in the tumor front and center and peripheral blood, while the proportion of Foxp3+ cells in the tumor infiltration front showed an increasing trend, indicating that the high expression of ICOSLG is likely to be involved in the occurrence of immunosuppression in the tumor microenvironment.

In the past few years, with the increasing use of immunotherapy, especially immune checkpoint inhibitors, there have been significant breakthroughs in the survival rate and prognosis of cancer patients (Miller et al., 2016). Immune checkpoint therapy enhances the anti-tumor effect in the tumor microenvironment by regulating the function of T cells (Sharma and Allison, 2015).In a preclinical drug experiment, it was found that the ICOS-ICOSLG pathway has a good clinical application prospect for immunotherapy of various tumors (Solinas et al., 2020). Our study found that the high expression of ICOSLG in HNSCC was positively correlated with multiple immune checkpoints. Including ICOS, CD96, PD-L1 (CD274), CSF1, CTLA, HAVCR2, IDO1, IL10, and PDCD1 were positively correlated.

In summary, we determined that ICOSLG was associated with the survival of OSCC patients and had a significant tumor-promoting effect. In addition, ICOSLG was positively correlated with Foxp3+ cells and negatively correlated with CD4^+^ T cells, indicating that ICOSLG is closely related to the immunosuppressive process in the tumor microenvironment. However, the specific mechanism and related molecular pathways of ICOSLG in OSCC for immune regulation in tumor microenvironment remain to be further studied. Future studies need to reveal the role of ICOSLG in tumorigenesis and development through immune regulation.

## Data Availability

The original contributions presented in the study are included in the article/Supplementary Material, further inquiries can be directed to the corresponding authors.
